# Rapid and Easy-Read Porcine Circovirus Type 4 Detection with CRISPR–Cas13a-Based Lateral Flow Strip

**DOI:** 10.3390/microorganisms11020354

**Published:** 2023-01-31

**Authors:** Jieru Wang, Xiaojie Zhu, Dongdong Yin, Chang Cai, Hailong Liu, Yuqing Yang, Zishi Guo, Lei Yin, Xuehuai Shen, Yin Dai, Xiaocheng Pan

**Affiliations:** 1 Anhui Province Key Laboratory of Livestock and Poultry Product Safety Engineering, Livestock and Poultry Epidemic Diseases Research Center of Anhui Province, Key Laboratory of Pig Molecular Quantitative Genetics of Anhui Academy of Agricultural Sciences, Institute of Animal Husbandry and Veterinary Sciences, Anhui Academy of Agricultural Sciences, Hefei 230031, China; 2 China Institute of Veterinary Drug Control, Beijing 100000, China; 3 China-Australian Joint Laboratory for Animal Health Big Data Analytics, College of Animal Science and Technology & College of Veterinary Medicine, Zhejiang A&F University, Hangzhou 311300, China; 4 Key Laboratory of Agricultural Animal Genetics, Breeding and Reproduction of Ministry of Education & Key Lab of Swine Genetics and Breeding of Ministry of Agriculture and Rural Affairs, Huazhong Agricultural University, Wuhan 430070, China

**Keywords:** porcine circovirus 4, CRISPR–Cas13a, RPA, LFD, rapid detection

## Abstract

First identified as a new circovirus in Hunan Province in China in 2019, porcine circovirus (PCV4) is now widely detected in other Chinese provinces and South Korea. In recent years, the virus has threatened pig health and operations in the pig industry. Hence, early PCV4 detection and regular surveillance are required to control the spread of infection and prevent collateral damage to the industry. Due to PCV4 being difficult to isolate in vitro, molecular detection methods, such as conventional PCR and real-time PCR, and serological assays are currently the main methods used for the detection of PCV4 infection. However, they are time-consuming, labor-intensive, and complex and require professional personnel. To facilitate rapid pen-side PCV4 diagnoses, we used clustered regularly interspaced short palindromic repeats (CRISPR) and Cas13a technology to develop a quick testing kit. Five recombinase-aided amplification (RPA) primer sets were designed based on the conserved PCV4-Cap gene nucleotide region, which were used to determine several key lateral flow strip (LFD) characteristics (sensitivity, specificity, and accuracy). The results showed that the RPA-Cas13a-LFD reaction could detect PCV4 within 1.5 h in genomic DNA harboring a minimum of a single copy. Furthermore, the assay showed good specificity and absence of cross-reactivity with PCV2, PCV3, or other porcine viruses. When we tested 15 clinical samples, a high accuracy was also recorded. Therefore, we successfully developed a detection assay that was simple, fast, accurate, and suitable for on-site PCV4 testing.

## 1. Introduction

Porcine circovirus (PCV) is a non-enveloped circular DNA virus that belongs to the *Circovirus* genus of the *Circoviridae* family [1]. PCV can induce severe damage in lymphoid tissue and cause immunosuppression [2,3]. The virus poses serious threats to global pig industry operations [4]. PCV was first discovered in 1974, and four species have been identified: PCV1, PCV2, PCV3, and PCV4 [5,6,7,8]. PCV4 was first identified in 2019 as a new circovirus in China [3]. Currently, PCV4 is widely distributed across several Chinese provinces and has been reported in Hunan, Henan, Shanxi, Jiangsu, and Guangxi, with prevalence rates varying from 3.3 to 25.4% [9,10]. Ge et al. conducted a nationwide PCV4 surveillance study and collected 1790 pig serum samples from 17 Chinese provinces (out of 34 provinces) [11]. The authors reported that sample seroprevalence was 44.0%. As a close neighbor of China, South Korea reported, in a nationwide survey covering six South Korean provinces (out of nine provinces), a PCV4 seroprevalence rate of 3.3% [12]. In contrast, Franzo et al. reported no PCV4 evidence in Spain or Italy [13].

Detection and surveillance are critical infectious disease prevention and control measures [14]. Therefore, early PCV4 detection and regular surveillance are key steps required to control virus spread and prevent potential damage to the pig industry. Currently, the most widely used PCV4 test methods are polymerase chain reaction (PCR) and real-time PCR (RT-PCR). However, these methods are time-consuming, labor-intensive, and complex and require professionals to operate complex equipment; therefore, they are not recommended for pen-side PCV4 diagnostics [3,15,16,17,18]. Consequently, new diagnostic tests must be developed, which can be easily performed by pig farmers and/or other stakeholders to provide rapid pen-side results.

*Leptotrichia wadei* (Lwa) clustered regularly interspaced short palindromic repeats (CRISPR)-associated protein13a (LwaCas13a), a CRISPR–Cas13a variant, has been rapidly developed for nucleic acid-based diagnostics by using its characteristic collateral activity [19]. CRISPR–LwaCas13a can be used to detect targeted RNAs and the collateral cleavage activity of nearby non-targeted RNAs, which can be detected with a fluorophore–quencher pair linked by an ssRNA, which fluoresces after cleavage by active LwaCas13 [20,21]. To achieve high sensitivity, current CRISPR diagnostics rely on the pre-amplification of target RNA for subsequent detection by a Cas protein. In the case of RNA-sensing LwaCas13a, this entails the conversion of RNA to DNA by reverse transcription, DNA-based amplification, and transcription back to RNA for detection by LwaCas13a [22]. The technology is characterized by a high sensitivity for RNA or DNA detection [21]. CRISPR–Cas technology has been successfully used in clinical rapid test kits for African swine fever virus [23], severe acute respiratory syndrome coronavirus 2 [24], PCV3 [25], *Mycobacterium tuberculosis* [26], *Staphylococcus aureus* [27], and Avian Influenza A [28].

In this study, we developed a PCV4-detection system by combining CRISPR–Cas13a, recombinase-aided amplification (RPA), and lateral flow strips (LFD). The PCV4 Cap gene was selected as the target to develop the CRISPR–Cas-based detection method. Our method could facilitate early and rapid PCV4 detection and control.

## 2. Materials and Methods

### 2.1. Plasmids and Viruses

Since it is difficult to isolate wild-type PCV4, the sequence of the PCV4-Cap gene (GenBank: OP221238) was synthesized, and sticky-end restriction enzymes were ligated for insertion into the pMD-19T vector (Takara, Dalian, China), resulting in a recombinant plasmid: pMD-19T-PCV4. PCV2 (GenBank: MK426833) was obtained from our laboratory repository. The recombinant plasmid pMD-19-PCV3-Cap was stored in our laboratory repository and was based on a PCV3 strain (GenBank: KY075995.1). Pseudorabies virus (PRV, Bartha-K61 vaccine strain), porcine reproductive and respiratory syndrome virus (PRRSV, CH-1R vaccine strain), and classical swine fever virus (CSFV, tissue culture origin) were purchased from the Harbin Veterinary Research Institute, China. Porcine parvovirus (PPV, PPVS-1A vaccine strain) was purchased from Qilu Animal Health Products Co., Ltd., China, and Japanese encephalitis virus (JEV) was purchased from Wuhan Keqian Biology Co., Ltd., China.

### 2.2. RPA Primer Design and crisprRNA (crRNA) Preparation

The amino acid sequences of PCV4-Cap genes from 38 PCV4 field strains were examined using multiple sequence alignments. A conserved 307 base pair nucleotide sequence template (Supplementary Material 1) was chosen to design RPA primers per design requirements. The T7 promoter sequence (GAAATTAATACGACTCACTATAGGG) was appended to the 5′ end of the RPA forward primer. Five primer sets were designed. Primers were synthesized by General Biological System Co., (Anhui, China) (Table 1).

In total, five Cas-13a crRNAs targeting the RPA amplification products of the Cap gene were designed. For crRNA preparation, crDNA templates were appended to the T7 promoter sequence and synthesized as primers by General Biological System Co., (Table 1). Two oligonucleotides were annealed to double-stranded DNA (ds-DNA) using annealing buffer for DNA Oligos (Beyotime, China), and then the ds-DNA was purified by gel extraction. In accordance with the instructions of the manufacturer, the ds-DNA was transcribed to crRNA using the HiScribe T7 Quick High Yield RNA synthesis kit (NEB, Massachusetts, USA). The crRNA was purified using the Agencourt RNAClean XP kit (Beckman Coulter, CA, USA) and stored at −80 °C. The FAM-N6-BIO probe (FB probe), used in fluorescent reporter assays, was synthesized by General Biological System Co., (Table 2).

### 2.3. Nucleic Acid Preparation

PCV2, PRV, PPV, PRRSV, JEV, and CSFV genomic DNA/RNA were extracted using a TIANamp Virus DNA/RNA Kit (Tiangen, China), in accordance with the instructions of the manufacturer. First-strand cDNA was generated using reverse transcription-PCR and a PrimeScript™ RT reagent kit with gDNA Eraser (TaKaRa, Shanghai, China). Then, DNA and cDNA samples were stored at −80 °C.

### 2.4. Evaluating and Optimizing RPA Reactions

RPA reactions were conducted using an RPA kit (DNA-LS01, LeSunBio, Wuxi, China), in accordance with the instructions of the manufacturer. Briefly, a 50 μL reaction comprised 25 μL buffer A, 13.5 μL nuclease-free water, 4 μL DNA, 1 μL RPA polymerase, 2 μL Cap-RPA-F (forward primer, 10 μM), 2 μL Cap-RPA-R (reverse primer, 10 μM), and 2.5 μL magnesium acetate. Reactions were incubated for 40 min at 37 °C. Primers were tested to select the best-performing primer pairs. Then, RPA reaction products were transferred to the CRISPR–Cas-13a cleavage assay.

### 2.5. Cas13a Nucleic Acid Detection

For Cas13a LFD, the Cas13a reaction system comprised 50 μL of 22.5 nM crRNA, 45 nM LwaCas13a (Magiltd, China), 125 nM FB probe, 0.25 μL RNase inhibitor, 1 mM dNTP, 0.4 μL T7 RNA Polymerase Mix (NEB, USA), and 1 μL RPA product. Cas13a detection was performed for 40 min at 37 °C. Then, CRISPR–Cas13a-LFD was diluted 10-fold in Hybridetect assay buffer (Magiltd) and loaded onto the LFD (Magigen, China). After 3–5 min incubation, results (specific line) were read and recorded.

### 2.6. PCV4 Analytical Sensitivity and Specificity by the RPA-Cas13a-LFD

The pMD-19T-PCV4 was prepared as follows. Briefly, the pMD-19T-PCV4 copy number was calculated based on the following formula:

$$Number\;of\;copies=\frac{Amount\;of\;DNA\;\left(ng\right)\times Avogadro^{\prime }s\;constant}{Length\;of\;DNA\;\left(bp\right)\times conversion\;factor\times Average\;mass\;of\;1bp\;of\;dsDNA}$$

Avogadro’s constant—This number (6.022 × 10^23^) represents the number of molecules in 1 mol. Conversion factor. The conversion factor (1 × 10^9^) is required to convert the value to ng. Average mass of 1 bp of dsDNA. The average mass of 1 bp of dsDNA is 660 g/mol. The concentration of pMD-19T-PCV4 was detected using Qubit dsDNA HS Assay Kits (Thermo Fisher Scientific, China), and then the amount of DNA was calculated. Length of DNA (3379 bp) is the length of pMD-19T (2692 bp) plus the length of Cap (687 bp).

We prepared 10-fold serial dilutions of pMD-19T-PCV4 templates (1.0 × 10^8^–1.0 × 10^0^ copies/mL) for RPA reactions, after which reaction products were subjected to Cas13a LFD assay. Assay data were used to calculate analytical sensitivity, while analytical specificity was assessed using genomic cDNAs or DNAs from a panel of pathogens, including PCV2, PCV3, PRV, PPV, PRRSV, JEV, and CSFV.

### 2.7. Evaluating RPA-Cas13a-LFD Tolerance to Serum Inhibitors

To evaluate RPA-Cas13a-LFD robustness in tolerating serum inhibitors, undiluted pMD-19T-PCV4 plasmids (359.2 copies/μL) and 1:0.001, 1:0.01, 1:0.02, 1:0.1, 1:0.5, 1:10, 1:20, 1:50, 1:100, and 1:200 dilutions were incubated with specific-pathogen-free pig serum as template. Reactions were analyzed using the RPA-Cas13a-LFD to evaluate amplification efficiencies with different serum dilutions in parallel.

### 2.8. Assay Validation Using Clinical Samples

Between 2020 and 2021, 15 clinical samples were collected from the Diagnostic Center, Anhui Academy of Agricultural Sciences, Anhui Province, China. Samples were used to confirm the applicability of the PCV4-specific Cas13a LFD for clinical diagnoses. Then, the results were compared with those obtained with RT-PCR, as described previously, which was run in parallel for the above clinical samples. The primers sequences: PCV4-F: 5′- CTGGAAGTGGAGGGTGT-3′; PCV4-R: 5′-ATGATGTCCTGGCAAAC-3′ (Zhang et al., 2020a).

### 2.9. Ethics Statement

The animal protocol was approved by the Ethics Committee (Animal Ethics Permit No: AAAS2021-3) of Anhui Academy of Agricultural Sciences, China. Verbal informed consent was obtained from animal owners.

## 3. Results

### 3.1. RPA Product Detection

**Figure 1:**
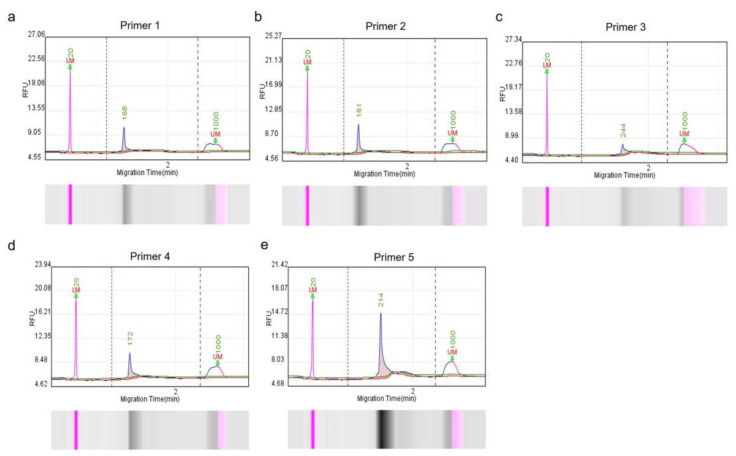
Capillary electrophoresis of RPA-amplified products, corresponding to five primer pairs (**a**–**e**). Products were analyzed using the Q-sep 100 automatic nucleic acid analysis system. The abscissa axis represents DNA fragment migration time, and the ordinate axis represents fluorescence signal intensity. The sample concentration is calculated by comparing the peak plot area of the standard concentration with the peak plot area of the test sample. Five primer pairs were used for RPA amplification.

Five RPA primer sets were designed based on the conserved nucleotide region of the Cap gene to ensure specific and efficient amplification. To screen out qualified primers, a Q-seq100 automatic nucleic acid analysis system was used to measure the fragment sizes and concentrations. The main peak fragment sizes of the corresponding RPA amplification products were similar to the theoretical sizes (Figure 1), and the product concentrations met the requirements of the subsequent detection studies (Table 3). Therefore, all primers were selected for further assessment.

### 3.2. Establishing the CRISPR–Cas13a-LFD

**Figure 2:**
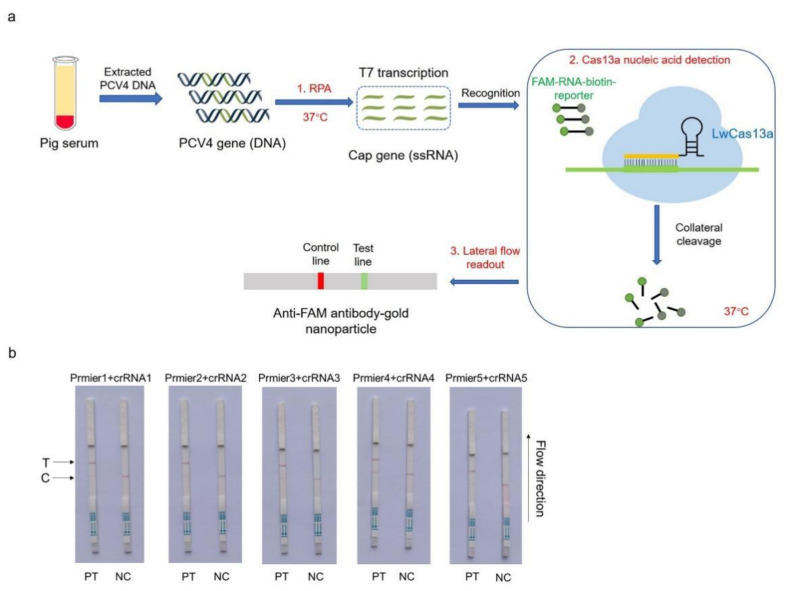
Establishing a CRISPR–Cas13a-LFD. (**a**) Schematic showing the Cas13a PCV4 test workflow. The PCV4-Cap gene was extracted from clinical pig serum, amplified by RPA, and transcribed to ssRNA to activate the Cas13a nuclease. Then, this nuclease recognized crRNA and cut off the reporter molecule. Finally, the reporter molecule appeared as a band on the test strip. RPA = recombinase-aided amplification; ssRNA = single-stranded RNA. (**b**) Analysis of different crRNAs by CRISPR–Cas13a-LFD. PT = pMD-19T-PCV4 standard plasmid; NC = negative control; T = test line; C = control line.

For on-site detection, a CRISPR–Cas13a-LFD assay was generated by combining RPA, LwaCas13a, and an LFD. The LFD was generated using a biotin-tagged FAM-RNA reporter. For the negative control, an anti-FAM antibody–gold nanoparticle was conjugated to the FAM-RNA-biotin reporter, and the conjugate was intercepted by a biotin ligand at the control line. For the positive control, the FAM-RNA-biotin reporter was cleaved, and the anti-FAM antibody–gold nanoparticle conjugate accumulated at the test line and then decreased at the control line. A schematic showing LFD is shown in Figure 2a. The practical workflow of this test on a farm includes three steps. Firstly, the PCV4-Cap gene, extracted from clinical pig serum, was amplified by RPA and transcribed to ssRNA to activate the LwaCas13a nuclease. Then, this nuclease recognized crRNA and cut off the reporter molecule. Finally, the reporter molecule appeared as a band on the test strip. Five Cas13a crRNAs were designed to target the RPA-amplified products of the PCV4 Cap gene. The results were shown on strips, and the crRNA2 and crRNA5 combination was selected for performing the CRISPR–Cas13a-LFD assays (Figure 2b).

### 3.3. CRISPR–Cas13a-LFD Analytical Sensitivity

**Figure 3:**
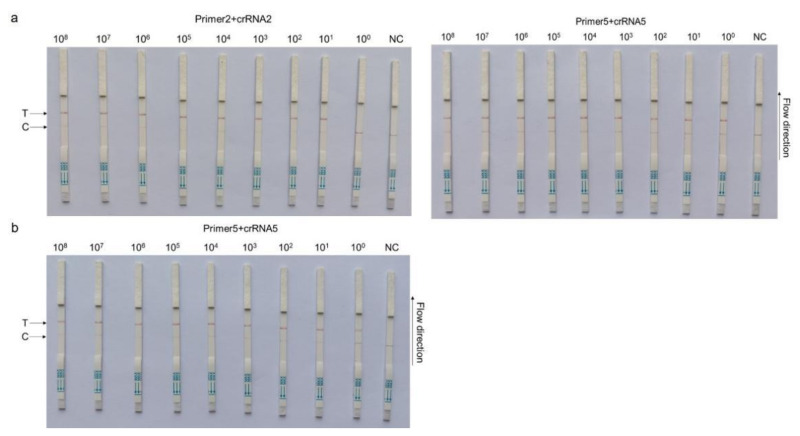
CRISPR–Cas13a-LFD sensitivity assays. A 10-fold serial dilution of pMD-19T-PCV4 was used as the detection template. (**a**) Sensitivity testing of different primer and probe combinations: primer2+crRNA2 (left) and primer5 + crRNA5 (right). (**b**) Sensitivity of the primer5 + crRNA5 combination after reaction system optimization. Detection reactions were performed using the following detection limits: 1 × 10^8^ copies/µL, 1 × 10^7^ copies/µL, 1 × 10^6^ copies/µL, 1 × 10^5^ copies/µL, 1 × 10^4^ copies/µL, 1 × 10^3^ copies/µL, 1 × 10^2^ copies/µL, 1 × 10^1^ copies/µL, and 1 × 10^0^ copies/µL. P = pMD-19T-PCV4 standard plasmid; NC = negative control; T = test line; C = control line.

Based on different primer pairs and probe combinations, the sensitivity of CRISPR–Cas13a-LFD was evaluated with a 10-fold serial diluted template at a concentration from 1 × 10^8^ copies/µL to 1 × 10^0^ copies/µL of the template. The combination of primer5 +crRNA5 had a higher sensitivity, for which the detection limit could reach 1 × 10^0^ copies/µL; primer2 + crRNA2 composition could reach the detection limit of 1 × 10^1^ copies/µL (Figure 3a). The primer5 + crRNA5 combination was selected for further clinical detection with 1 × 10^0^ copies/µL, which was the ultimate limit of detection for this method (Figure 3b).

### 3.4. CRISPR–Cas13a-LFD Analytical Specificity

**Figure 4:**
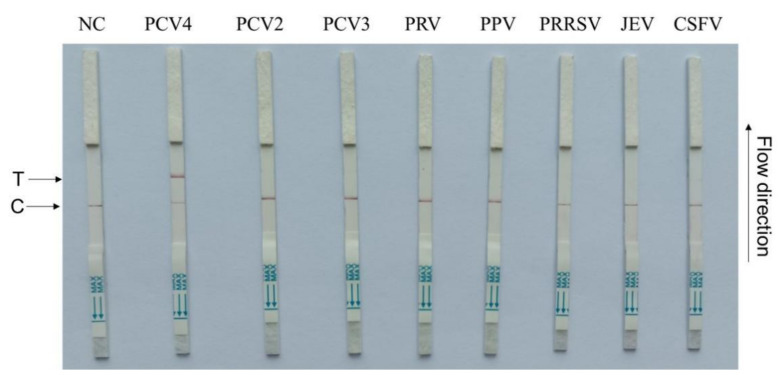
CRISPR–Cas13a-LFD specificity assays. Porcine circovirus 4 (PCV4), PCV2, PCV3, pseudorabies virus (PRV), porcine parvovirus (PPV), porcine reproductive and respiratory syndrome (PRRSV), Japanese encephalitis virus (JEV), and classical swine fever virus (CSFV)). NC = negative control; T = test line; C = control line.

To test CRISPR–Cas13a-LFD specificity, seven swine pathogens, including PCV2, PCV3, PRV, PPV, PRRSV, JEV, and CSFV, were used. The primer5 + crRNA5 combination showed that positive LFD signals were identified only for PCV4, while negative signals were observed for all the other swine pathogens (Figure 4). Therefore, our assay demonstrated high PCV4 specificity.

### 3.5. Evaluating RPA-Cas13a-LFD Tolerance to Serum Inhibitors

**Figure 5:**
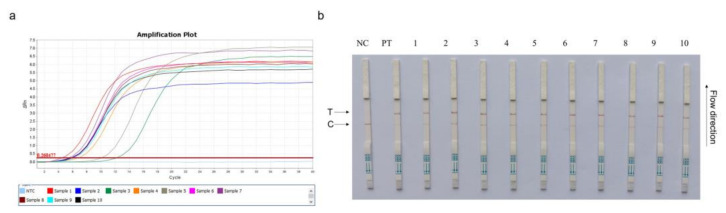
Spiked-in serum inhibitor interference in RT-PCR (**a**) and CRISPR–Cas13a-LFD (**b**) assays. NC = negative control; PT = pMD-19T-PCV4 plasmids (359.2 copies/μL), 1: serum (S): PT = 1:0.001, 2: S:PT = 1:0.01, 3: S:PT = 1:0.02, 4: S:PT = 1:0.1, 5: S:PT = 1:0.5, 6: S:PT = 1:10, 7: S:PT = 1:20, 8: S:PT = 1:50, 9: S:PT = 1:100, and 10: S:PT = 1:200. T = test line; C = control line.

The inhibitory effects of serum in the RPA-Cas13a-LFD and RT-PCR assays are shown in Figure 5. Serum addition to RPA-Cas13a-LFD and SYBR Green-based RT-PCR assays (Zhang et al., 2020a) failed to inhibit reactions at any concentration.

### 3.6. Clinical Sample Detection Using CRISPR–Cas13a-LFD

**Figure 6:**
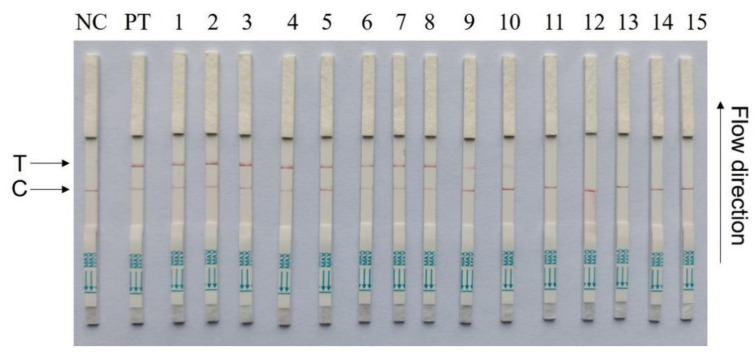
Clinical sample detection by CRISPR–Cas13a-LFD assays. Fifteen samples were used for testing. NC = negative control; PT = pMD-19T-PCV4 plasmids; T = test line; C = control line.

To validate the CRISPR–Cas13a-LFD reliability for PCV4 detection in clinical samples, 15 tissue samples collected from different farms were detected by CRISPR–Cas13a-LFD and RT-PCR assays (Figure 6). Ten tissue samples were PCV4-positive, and five samples were PCV4-negative, as determined by Cas13a lateral flow detection. RT-qPCR displayed the same results as Cas13a lateral flow detection in clinical samples (Table 4). These data indicated that CRISPR–Cas13a-LFD detection could be used in clinical samples, which does not require expensive equipment.

## 4. Discussion

Recently, Niu et al. successfully rescued PCV4 from an infectious clone and found that PCV4 was distributed widely in the tissues of infected piglets [29]. Nevertheless, no isolated PCV4 strain is available yet [30]. Since PCV4 was first discovered in China in 2019, several outbreaks have been reported across many provinces and other countries [11,31]. Besides in domestic pigs, PCV4 has also been identified in wild boars and dairy cows [32,33]. Rapid and accurate disease diagnoses are vital to prevent disease spread; therefore, PCV4 monitoring in disease-free countries/zones is warranted. The ideal diagnostic methods should be inexpensive, accurate, fast, and easy to use and should not require specialized equipment or personnel. However, the current PCV4 methods lack these critical advantages; thus, specific and sensitive PCV4-detection methods that meet these demands are imperative. To this end, we established an RPA-Cas13a-LFD assay that rapidly, conveniently, and sensitively detected PCV4.

RPA-CRISPR specificity depends on the number of mismatches in the crRNA-targeted nucleotide region; therefore, we compared and analyzed PCV4 genomes [23,34]. From the alignment results, the PCV4 Cap gene was selected as the target gene, and five RPA primer sets were designed and amplified. Of the five primer–probe combinations, based on preliminary detection data, we selected the most sensitive for optimization. Subsequently, assay analytical sensitivity reached the single-copy level for the plasmid. Zhang et al. developed a real-time TaqMan PCR assay to detect PCV4 using a minimum of 22 copies [3], and Chen et al. established a quadruplex real-time PCR for all species of PCV strains with the detection limit of 28 copies/μL [30]. Two SYBR Green-based real-time PCR assays were developed by Zhang [3] and Hou et al. [35] that exhibited sensitivity as low as 3 and 67.7 copies/μL, respectively. The sensitivity of the loop-mediated isothermal amplification (LAMP) assay developed by Li et al. [36] was shown to be 10 copies. In contrast, the RPA-Cas13a-LFD system described in the present study was able to detect PCV4 with even a single copy, demonstrating its high sensitivity relative to previous methods and its ability to be used for rapid and specific pen-side testing.

In our study, detecting LwaCas13a in combination with LFD facilitated application in both laboratory and field settings. Moreover, combining RPA and crRNA-specific sequence identification ensured that the LwaCas13a detection method was more specific [22]. The assay showed no cross-reactivity with seven other swine viruses, thereby showing excellent specificity, which is vital for clinical testing.

Additionally, to verify CRISPR–Cas13a-LFD PCV4 reliability for clinical diagnostics, 15 clinical samples were verified and compared with a reported RT-PCR assay, which showed that both methods had coincidence percentage rates of 100% for PCV4 sample detection. However, this study has some limitations: the number of clinical samples was simply too small, and, in particular, there was a lack of different types of tissue samples to check the related sensitivity.

The test is suitable for remote farms that lack sophisticated instruments and inexperienced inspectors. After DNA extraction from clinical porcine serum, the PCV4-Cap gene was amplified by RPA and transcribed into ssRNA within 40 min in a 37 °C water bath to activate the LwaCas13a nuclease. Nucleases then recognize the crRNA and cleave the reporter gene within 40 min in a 37 °C water bath. Finally, the reporter molecule appears as a band on the strip within 5–10 min. In practice, the throughput of this test depends on the size of the water bath, and the most suitable range of use is no more than 50 at a time.

We developed a highly sensitive and specific PCV4 Cap gene-detection method. When compared with conventional methods, our RPA-Cas13a-LFD assay was simple, fast, accurate, and suitable for on-site testing. Therefore, our assay has potential applications for detecting and controlling PCV4 in the field. All authors have read and agreed to the published version of the manuscript.

## Figures and Tables

**Table 1:** Recombinase-aided amplification (RPA) primer sequences.

Name		Sequence (5′-3′)
Primer 1	Cap-RPA-F1 Cap-RPA-R1	TGCTGTGGTTTGCCAGGACATCATAAGTTT TCCCATTTGCATATTACCGGATCAGAAAGG
Primer 2	Cap-RPA-F2 Cap-RPA-R2	GACATCATAAGTTTGGTTTTTCCCTTCCCCC TTTACAGCCTCCCATTTGCATATTACCGGAT
Primer 3	Cap-RPA-F3 Cap-RPA-R3	TCATAAGTTTGGTTTTTCCCTTCCCCCACATAG CACGCCCTCTTGGAACGTTGGACATTACGATTT
Primer 4	Cap-RPA-F4 Cap-RPA-R4	CTGCTGCTGTGGTTTGCCAGGACATCATAAGTTT AGCCTCCCATTTGCATATTACCGGATCAGAAAGG
Primer 5	Cap-RPA-F5 Cap-RPA-R5	TTTTTCCCTTCCCCCACATAGTCTCCATCCAGTT GCCCTCTTGGAACGTTGGACATTACGATTTCAAA

**Table 2:** Cluster regularly interspaced short palindromic repeat RNA (crRNA) sequences.

Name	Sequence (5′-3′)
crRNA1	AACTGGATGGAGACTATGTGGGGGAAGG
crRNA2	GAAAGGTCAAAGTCGAATTTCTGCCACT
crRNA3	TTACAGCCTCCCATTTGCATATTACCGG
crRNA4	AACTGGATGGAGACTATGTGGGGGAAGG
crRNA5	TTACAGCCTCCCATTTGCATATTACCGG
FAM-N6-BIO probe	/56-FAM/mArArUrGrGrCmAmArArUrGrGrCmA/3 Bio/

**Table 3:** Amplified products as detected by the Q-sep 100 assay.

Primer Name	Peak Fragment Size (bp)	Theoretical Fragment Size (bp)	Product Concentration (ng/µL)
PCV4 Primer1	168	162	1.44
PCV4 Primer2	161	155	1.56
PCV4 Primer3	244	231	1.09
PCV4 Primer4	172	170	1.7
PCV4 Primer5	214	216	3.37

**Table 4:** Clinical sample detection results using the Cas13a lateral flow detection assay.

Assay	Number of Samples	
	Positive	Negative
RT-PCR	10	5
Cas13a lateral flow detection	10	5

## Data Availability

The original contributions to the study are included in the article/supplementary material. Further inquiries can be directed to the corresponding author.

## Supplementary Materials

The following supporting information can be downloaded at: https://www.mdpi.com/article/10.3390/microorganisms11020354/s1. The conserved 307 base pair nucleotide sequence of PCV4-Cap gene.
