# Supplementary material for: Rapid and Easy-Read Porcine Circovirus Type 4 Detection with CRISPR–Cas13a-Based Lateral Flow Strip
Source: Microorganisms. 2023 Jan 31;11(2):354. doi: 10.3390/microorganisms11020354 (PMC9967505; doi:10.3390/microorganisms11020354)
Supplement: Supplementary file 1 [file microorganisms-11-00354-s001.zip › microorganisms-2163008-supplementary.pdf]

Supplementary Material

1 Supplementary Data 1.

The conserved nucleotide region of the PCV4 Cap gene：

5’TTTGGAGTGAAATAGCGACTGTGTCTAGCAATATTGGTGAAACCATGCCTGCTGCTGT
GGTTTGCCAGGACATCATAAGTTTGGTTTTTCCCTTCCCCCACATAGTCTCCATCCAGTTG
TATAGCAGTGCTAGAGTAAGTCCTATTACTGTTAATGCCATTTAGTGGCAGAAATTCGAC
TTTGACCTTTCTGATCCGGTAATATGCAAATGGGAGGCTGTAAAGGTTTACGATCGTTCC
CGGTCCTTTTGGGATAAAGTCCTTCAGTTTGAAATCGTAATGTCCAACGTTCCAAGAGGG
CGTGGAAA-3’
